# Improving bioinformatics software quality through incorporation of software engineering practices

**DOI:** 10.7717/peerj-cs.839

**Published:** 2022-01-05

**Authors:** Adeeb Noor

**Affiliations:** Department of Information Technology, Faculty of Computing and Information Technology, King Abdulaziz University, Jeddah, Saudi Arabia

**Keywords:** Documentation, Bioinformatics, Education, Testing, Integration, Requirements, Software engineering, Reproducibility

## Abstract

**Background:**

Bioinformatics software is developed for collecting, analyzing, integrating, and interpreting life science datasets that are often enormous. Bioinformatics engineers often lack the software engineering skills necessary for developing robust, maintainable, reusable software. This study presents review and discussion of the findings and efforts made to improve the quality of bioinformatics software.

**Methodology:**

A systematic review was conducted of related literature that identifies core software engineering concepts for improving bioinformatics software development: requirements gathering, documentation, testing, and integration. The findings are presented with the aim of illuminating trends within the research that could lead to viable solutions to the struggles faced by bioinformatics engineers when developing scientific software.

**Results:**

The findings suggest that bioinformatics engineers could significantly benefit from the incorporation of software engineering principles into their development efforts. This leads to suggestion of both cultural changes within bioinformatics research communities as well as adoption of software engineering disciplines into the formal education of bioinformatics engineers. Open management of scientific bioinformatics development projects can result in improved software quality through collaboration amongst both bioinformatics engineers and software engineers.

**Conclusions:**

While strides have been made both in identification and solution of issues of particular import to bioinformatics software development, there is still room for improvement in terms of shifts in both the formal education of bioinformatics engineers as well as the culture and approaches of managing scientific bioinformatics research and development efforts.

## Introduction

Along with the many recent advancements in the life sciences, which include, but are not limited to, advancements in drug design processes and other innovations under the umbrella of biochemistry, the field of bioinformatics has arisen with scientists collecting, integrating, analyzing, and interpreting large life science datasets. The Oxford English Dictionary defines "(molecular) bio-informatics as the conceptualization of biology in terms of molecules (in the sense of physical chemistry) and the application of “informatics techniques” (derived from disciplines such as applied mathematics, statistics, and computer science) to understand and organize the information associated with these molecules on a large scale. Bioinformatics can be seen as an management information system for molecular biology with many practical applications. According to the National Institutes of Health (NIH), bioinformatics can be described as any research, development, or application that could help scientists visualize, store, analyze, and/or organize biological data (Bayat, 2002). In the healthcare industry, application of bioinformatics techniques has seen the electronic health records annotated with disease diagnoses and medications (Hoffman, 2007) mapped to precision medicines (Tenenbaum, Shah & Altman, 2021) with worldwide development and adoption (Noor, 2019a). In a different direction, computational biology has been applied with great precision to addressing specific tasks, such as the discovery of genes (Althubaiti et al., 2019). There are other examples of the development of specialized software for solving life science problems, such as the prediction of drug–drug interactions (Noor et al., 2017), the design of novel drugs (Assiri & Noor, 2020), and the discovery of new treatment methods for diseases (Noor & Assiri, 2021). These accomplishments and many others have been made possible through advances in computation technology and the growing availability of massive life science datasets (Gupta, Gangotia & Mani, 2021).

Despite the many existing and upcoming applications of bioinformatics that demonstrate great potential for healthcare advancement, bioinformatics scientists themselves have yet, for the most part, to evolve along with the bioinformatics field in terms of acquiring refined skills for developing high-quality, maintainable software (Lawlor & Sleator, 2021). In a review of two popular bio-sequence alignment software packages for bioinformatics, FAST and BLAST (Aryal, 2019; Leimeister et al., 2014; Cashman et al., 2018) concluded that both tools would greatly benefit from improved flexibility, usability, and user-friendliness. Sanders & Kelly (2008), Oulas et al. (2019) and Lawlor & Sleator (2021) reinforced the notion that the complex bioinformatics discipline and its increasing depth and volume of applications necessitate the development of reliable and maintainable software for use and expansion by bioinformatics professionals. In a similar vein, Borgman & Bourne (2021) provided a related discussion of the issues of software data management. Clearly, unreliable and/or unmaintainable software pose a real hindrance to the success and expansion of the application of bioinformatics approaches.

In order to address the issue, it must be recognized that bioinformatics software packages are often conceptually different, ranging from throw-away scripts to one-off applications to reusable platforms, which can greatly affect the software engineering practices that should be adopted. For instance, scientific bioinformatics software has been defined as software developed in the academic setting for the solution of a domain issue (Howison et al., 2015). The development of such software faces many challenges in terms of documentation, testing, continuous integration, and containerization for portability.

One of the fundamental, exacerbating factors to the development of high-quality bioinformatics software is a lack of initiatives in formal education supporting the instillation of software engineering principles into existing and upcoming bioinformatics professionals. This is supported by studies, such as Georgeson et al. (2019), that indicate bioinformatics engineers often apply *ad hoc* methodologies when developing scientific bioinformatics software. The situation is compounded by the fact that traditional software engineers are usually unfamiliar with the complexities of the life sciences (Koch & Jones, 2016), resulting in bioinformatics engineers needing to develop their own software. Of the problems associated with the development of bioinformatics scientific software, gathering and defining system requirements, producing proper documentation, and eliminating code redundancy are frequent (Segal, 2007) because self-study of coding, rather than formal software development, is most common among bioinformatics engineers (Weston, 2004; Attwood et al., 2019).

Studies in bioinformatics historically began with informal workshops and training then progressed to certification and finally Ph.D. programs (Ranganathan, 2005). Unfortunately, while many of these programs have deeply focused on biological courses and shallowly focused on software development courses with many of the popular programs offering few software engineering courses specific to addressing the complexities of bioinformatics. According to the study “Software Engineering Education for Bioinformatics” (Umarji et al., 2009), only two out of 79 programs considered at popular higher-education institutions within the United States included some type of software engineering course. The study also demonstrated that 85% of bioinformatics students learned to code through self-study. Similar situations have occurred outside of the United States where significant national resources were earmarked for the development of scientific-educational programs, such as Vision 2030 in the Kingdom of Saudi Arabia, yet few opportunities exist for formal studies in bioinformatics at the tertiary level (Noor, 2019b). This phenomenon is further supported by quantities of research showing this lack of education and training among bioinformatics engineers (Baxter et al., 2006; Hannay et al., 2009; Merali, 2010; Joppa et al., 2013; Wilson, 2014; Wilson et al., 2014; Lawlor & Walsh, 2015; List, Ebert & Albrecht, 2017; Mulder et al., 2018). Additionally, a study was conducted that emphasizes the importance of collaboration/education among bioinformatics and software engineers regarding the development of reliable software (Lawlor & Sleator, 2021). Given the body of research, the need to incorporate formal software engineering principles into the curricula of bioinformatics is evident if there is to be any tangible hope of steady, scientific, efficient progress in the future of bioinformatics.

## Rationale

There are many established norms and patterns that are detrimental to the production of high-quality, maintainable scientific bioinformatics software, such as funding patterns that reward new development and preclude reuse, as well as the tendency of third-party code to lack sustainability and support for adoption. Issues often occur with bioinformatics software development because of the sophisticated research questions driving the software development as well as constraints involved in the associated projects such as time and budget. Most bioinformatics engineers benefit from incorporating software engineering skills, regardless of the scale or type of software to be developed. Such skills include documentation, testing, integration, and containerization for portability. Lawlor & Sleator (2021) confirmed the generally low-quality state of bioinformatics software development and emphasized the importance of collaboration among bioinformatics and software engineers for producing reliable software. A recent study (Brandies & Hogg, 2021) emphasized ten rules for undertaking bioinformatics software development.

In light of these efforts, this study presents a review of the relevant literature and suggests that cross-education of bioinformatics engineers in software engineering appears to be the most appropriate course of action for improving scientific bioinformatics software. The findings of this work identify challenges and opportunities of bioinformatics research and development efforts for addressing life science problems that could prove beneficial to policymakers concerned with science who are looking for tools and strategies to enhance the quality of software development. The following section presents the search strategy for extracting relevant articles in the systematic review. The third section discusses the state and possibilities of key software development challenges identified by the literature review, which include requirements gathering, documentation, testing, and integration. The fourth section presents practical conclusions and discussion based on the findings of the work for the purpose of developing higher-quality bioinformatics software.

## Search Strategy

A systematic review of literature related to the aim of this work was undertaken in conjunction with a review of the challenges faced by bioinformatics engineers. The results of this effort indicate many of the presupposed solutions to the fundamental concerns. The process recommended by Briner & Denyer (2012) and some attributes of the Preferred Reporting Items for Systematic Reviews and Meta-Analyses (PRISMA) statement were implemented for providing a reproducible, scientific, and transparent literature review of software engineering and bioinformatics cross-education. The entire methodological approach is encompassed in the following steps:

- Identification of need, preparation of a proposal, and development of a protocol for review
- Identification of research, selection of studies, assessment of quality, and acquisition/extraction/synthetization of data
- Reporting of the results for the review

**Figure 1:**
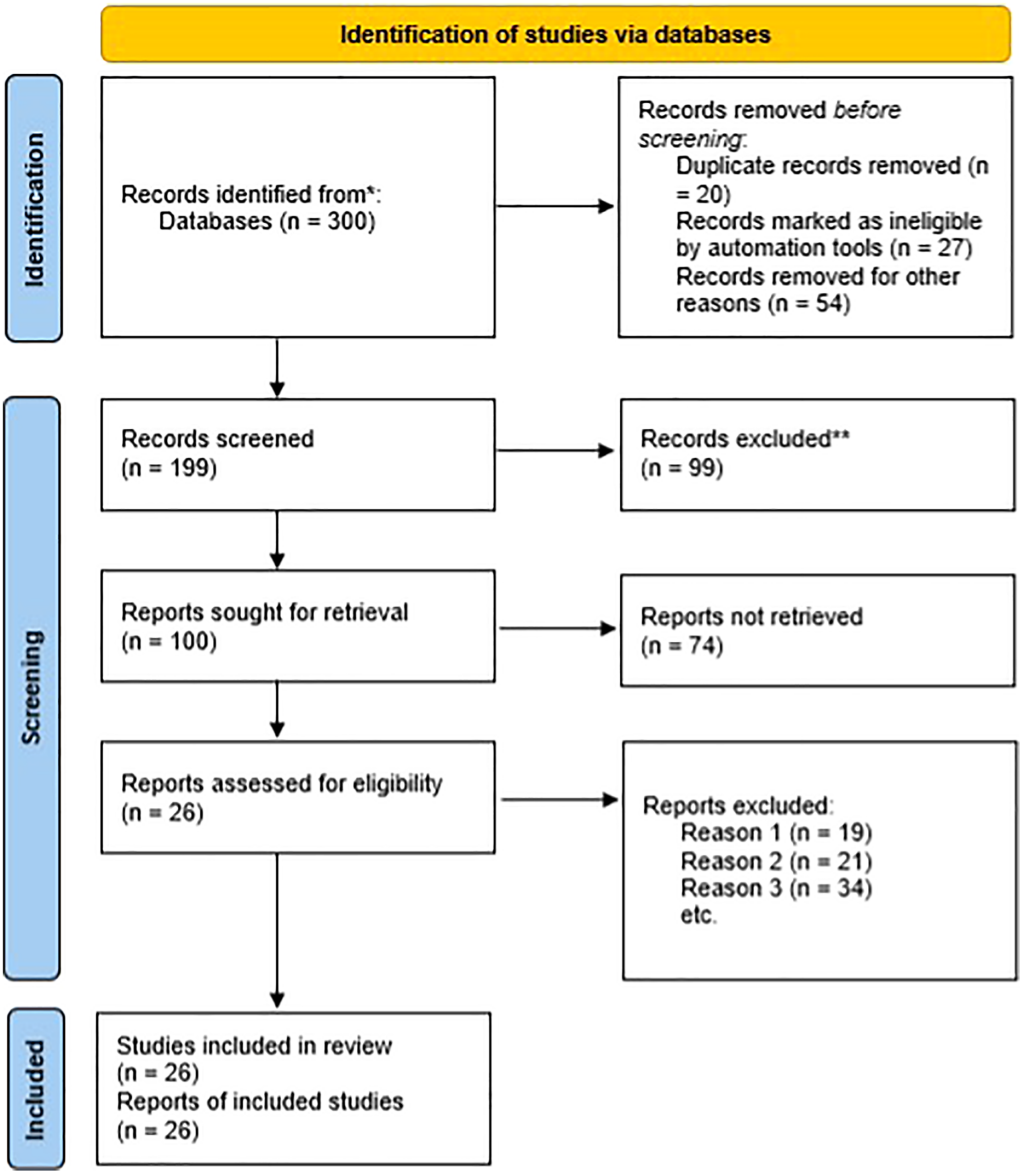
Search strategy based on PRISMA.

A systematic literature search was conducted for addressing the primary research question during January of 2019 of literature ranging in publication from 1990–2019. The findings, which served as a foundation, were augmented with research of additional developments published through July of 2021. The primary scientific database used was Scopus in which the terms “bioinformatics”, “engineering”, and “education” were searched across all available articles titles. The relevant articles selected for reference were additionally searched based on the snowball effect. Electronic searches were performed for grey literature, which includes unpublished research commissioned by private institutions, public institutions, and government entities. The first 300 article hits from Google were considered of as a sample of published grey literature after excluding unpublished reports, abstracts, review papers, and conference reports. Complete articles were retrieved and evaluated for relevance when abstracts were not available (Fig. 1). All reports and articles fulfilling the inclusion criteria were documented for bibliographical purposes with the data being categorized into emerging software challenges based upon independently-conducted content analyses.

## Challenges and Their Opportunities

From the literature search, four central areas of challenge in software development arose: requirements, documentation, testing, and integration. These challenges focus around reproducibility, which is critical for scientific bioinformatics software as well as scientific software in general, for that matter. Significant weakness in any of the four identified areas can lead to unmaintainable and potentially unusable software. Each area has had advancements and developments that have led to actual or potential quality and longevity improvements in scientific bioinformatics software. The adoption of certain processes and systems for the development and management of scientific bioinformatics software has demonstrated benefits for multiple of the areas.

### The challenge of requirements

According to Segal (2008), scientists usually lie with requirements because they are either unaware of their meaning or cannot properly define them. This is directly at odds with the practice in software engineering of gathering requirements to determine the intended purpose of the software to be developed. To be more complete, the definition process includes surveying the data, studying the current system, identifying business requirements, researching alternatives, and proposing a system (Bourgeois, 2014). Accurate definition of requirements ensures that the system to be developed will be unambiguous, testable, traceable, and measurable (Hay, 2003). Making the situation yet more challenging, in the context of bioinformatics, software requirements are naturally dynamic (Rother et al., 2012). With life science datasets continuing to yield new information and directions, the recording of novel occurrences requires a functional and flexible system (Hauth & Burger, 2008; Abdurakhmonov, 2016). Moreover, a study by Segal & Morris (2008) indicated that software requirements for scientific functions are different from those for business functions. Their study described a model of software developed by bioinformatics engineers wherein the bioinformatics engineers did not appreciate the process of gathering requirements for software engineers because they already knew the requirements of the scientific problem. The authors came to the conclusion that the bioinformatics engineers were often untruthful in discussions about their needs because they lacked awareness or clarity of definition of the requirements. In a twist, a different study by Rother et al. (2012) showed that bioinformatics engineers were often asked only for functional requirements, preventing associated software engineers from gathering adequate information about the system itself. This seems a bit ironic given that two other studies found that bioinformatics engineers working on projects of various sizes assigned software requirements the lowest rank in terms of project importance (Hannay et al., 2009; Garousi et al., 2019). Perhaps what is missing here is establishment of effective process more than rigorous enforcement of requirements.

Due to bioinformatics being an investigative discipline, the gathering of software requirements might benefit from the Agile approach, which can be summarized as “principles that emphasize building working software people can get their hands on quickly rather than spending a lot of time writing specifications up front” (Duka, 2013). This application of Agile is supported by Kane et al. (2006) who stated that the Agile method is balanced effectively due to the iterative and exploratory nature of the scientific inquiry. Similarly, Verma et al. (2013) proposed an approach to resolve the challenge of gathering bioinformatics software requirements based on the application of four methodologies: UP, domain engineering, SSADM, and Agile. O’Connor et al. (2008) recommended the sharing of customized analyses through the Generic Model Organism Database (GMODWeb), an integrated workflow system. A few examples of software packages that have been developed with best practices in mind include Tophat (Trapnell et al., 2012), BioPerl project (Leprevost et al., 2014), and Bowtie (Kishchuk et al., 2018). At a basic level, the usage of requirements tracking platforms (*e.g*., Jira: https://www.atlassian.com/software/jira) and development platforms (*e.g*., GitHub: https://github.com/) can help with definition of requirements, effective communications, and successful collaboration of bioinformatics engineers towards the development of high-quality software.

### The challenge of documentation

Proper documentation is often essential for maintaining custom software, especially software encompassing the integral complexities of bioinformatics research. Unfortunately, bioinformatics engineers often do not focus on documentation (Karimzadeh & Hoffman, 2018). Without documentation, it can be difficult if not impossible to understand the impetus for certain software design decisions. Such documentation can often take the form of a technical manual or a user manual. A technical manual describes the underlying functionality of the software system. A user manual, as its name implies, helps end-users of a software system properly leverage the software (Chimalakonda & Venigalla, 2020; Venigalla & Chimalakonda, 2021). Additionally, a user manual can provide some indication of the purpose or input-output requirements of the software.

When it comes to bioinformatics software development, the documentation process is not only necessary for the increasing number of end-user developers (Letondal & Mackay, 2004) but also the growing number of research projects analyzing and interpreting massive life science datasets which require the development of bioinformatics software (Brandies & Hogg, 2021). When Umarji et al. (2009) questioned a group of bioinformatics engineers, 90% of them indicated that documentation was valuable. The work of Taschuk & Wilson (2017) corroborated the need for documentation. Katerbow et al. (2018) also highly recommended documentation when developing scientific software. Hoda, Salleh & Grundy (2018) described the importance of knowledge in documentation and how it aided software development when using the Agile method. This conclusion chains into the work of Kane et al. (2006) who found that the Agile method can be applied across different bioinformatics tools utilizing extensive quantities of data through the creation of data types and formats to create a functional workflow. Further, documentation helped to embed domain knowledge in source code and improve domain-specific language to promote word sense disambiguation in the software (Ecale Zhou et al., 2019). Documentation can also provide both developers and end-users with supplementary information such as benchmarking results (Ison et al., 2019). There have been other efforts made in improving documentation for software developed for scientific bioinformatics.

According to Ison et al. (2016), improved documentation can likely assist in sustaining stable sharing mechanisms for metadata, particularly for institutional collections (*i.e*., Institute of France Bioinformatics (IFB)) and specialized registries (*i.e*., BioContainers), as well as improve community standards through OpenAPI and many biological databases (*i.e*., Web APIs).

To aid in the creation and formatting of documentation to improve its effectiveness, templates can be constructed for each type of documentation (*e.g*., user guides and system requirements) to that will give guidance to bioinformatics engineers (Karimzadeh & Hoffman, 2018). Furthermore, the use of the Agile methodology for effective documentation and software functionality development helps meet the information needs of the user while simultaneously allowing the application of different tools, such as automated workflow composition (Harris et al., 2019). Automated workflow, which refers to process design, execution, and automation based on workflow rules, can make the curation of bioinformatics software easier by employing different curation tools to derive information from standardized environments (*e.g*., Galaxy) or other code repositories (*e.g*., GitHub) to help identify and correct inconsistent information. Proper documentation is particularly important for the bioinformatics community and the international infrastructure of life science research when it comes to explaining and evaluating discoveries and building upon the research of others (Orengo et al., 2020).

### The challenge of testing

Software testing assesses the functionality of a software application to determine if predetermined requirements have been met and the resulting product performs to expectations (Patton, 2006). Each step of the software life-cycle is testable. Examples of types of software testing include validation testing, function testing, and unit testing. For scientific bioinformatics software, testing has often proven the most ambiguous and challenging task in the development process due to the software frequently being either data-driven or algorithm-driven, which can prove difficult to validate or verify. This is a type of oracle test problem where given a particular set of inputs for a system, there exists a “challenge of distinguishing the corresponding, desired, correct behavior from potentially incorrect behavior” (Barr et al., 2014).

This challenge can prove quite detrimental for bioinformatics engineers lacking software engineering skills who develop *ad-hoc* software for specific projects/tasks (Georgeson et al., 2019). The resulting *ad-hoc* used to determine software performance at an early stage, especially without proper documentation, planning, or processes, yields results that tend to be unstructured and inconsistent, frequently relying on the knowledge and memory of the expert doing the testing (Chhabra, 2012). When developing bioinformatics software, developers must have an in-depth understanding of the knowledge domain, whereas in other areas of software development (*e.g*., ERP systems), developers often do not need such extensive knowledge (Amershi et al., 2019). Furthermore, the interdisciplinary nature of bioinformatics requires its professionals to possess a wide range of abilities, resulting in bioinformatics engineers being more likely to be end-users (Allen & Mehler, 2019). To address testing issues (Soergel, 2014) argued that multiple sets of eyes are required in order to validate bioinformatics software and any research results that it may deliver. Hardware issues present a somewhat unique challenge for testing bioinformatics software. Limited hardware infrastructure can greatly restrict solution options for enormous life science datasets, affecting scalability, latency, performance of data infrastructures, and processing the data itself (Khan et al., 2014).

There have been some efforts to address testing of scientific bioinformatics software. Chen et al. (2009) proposed a metamorphic testing technique that tests properties from software outputs and end-users in parallel rather than using the traditional input-output testing approach. Kamali et al. (2015) surveyed current efforts in bioinformatics software testing and concluded that more research is needed. Lundgren & Kanewala (2016) experimented with subtle faults in testing bioinformatics software by applying pseudo-oracle and metamorphic tests on a genome alignment tool (BBMap). They found that metamorphic tests were effective in detecting subtle faults. Studies in scalability and validation have been performed and extensively reviewed with solutions such as cloud-based (Troup et al., 2016), divide-and-conquer, and multiple execution being examined for large bioinformatics datasets (Yang, Troup & Ho, 2017). Georgeson et al. (2019) created a command-line tool for developing scientific bioinformatics software that provides integrated unit and integration testing capabilities.

Version control systems can also aid in the testing of bioinformatics software through the use of certain tools, such as regression tests. Many free services exist, such as thefreecountry.com and Github.com, that provide version control solutions with tools that could prove helpful for bioinformatics software testing. Mulder et al. (2018) strongly encouraged bioinformatics engineers to utilize version control systems in order to maintain code quality and availability for the community. This also has the added benefit of exposure of the utilized software management processes to experienced software developers who can provide professional feedback and assistance. The Agile methodology can be applied when managing a project to divide the project into several different phases that involve constant collaboration with stakeholders and continuous enhancement at early stages. Two studies of software development using Agile methodology proved that scientists, in general, were well-satisfied with the resulting software testing (Umarji et al., 2009; Dingsøyr et al., 2018). A testing approach that could prove to be very effective involves scientists exposing all relevant source code during publication processes. This would not only allow for third-party validation, but also bolster testing results for reproducibility. Numerous efforts such as the Bioconductor (Gentleman et al., 2004) and OpenEBench (https://openebench.bsc.es) platforms have been undertaken for this purpose (Capella-Gutierrez et al., 2017).

### The challenge of integration

Being able to replicate and reproduce scientific results has progressively grown in significance for most of academic disciplines (Stodden, Borwein & Bailey, 2013; Howison et al., 2015; Miyakawa, 2020). For bioinformatics software, integration plays an important role in reproducibility. Gulledge (2006) concluded that “integration implies that all relevant data for a particular bounded and closed set of business processes are processed in the same software application.” According to Barker & Thornton (2004), integration has been proven to be the most important challenge for bioinformatics software over the next decade. However, the vast diversity of life science datasets, including genome sequences, literature, and software, can make integration seemingly impossible (Hannay et al., 2009; Lapatas et al., 2015). Problems can be faced when trying to handle the challenges of integrating data of differing qualities while incorporating sources of disseminated information (Miyakawa, 2020). Platform and system peculiarities can pose particular problems for integration due to interoperability concerns between them (Karasavvas, Baldock & Burger, 2004; Koru et al., 2007; Chilana, Palmer & Ko, 2009; Mangul et al., 2019). Differences between libraries from one operating system or version to another can result in varying outcomes when making system calls with software. If bioinformatics software is developed on a UNIX platform, for example, a developer will incorporate some libraries in the code that will enable it to operate on the UNIX platform (Gentleman et al., 2004). Public sharing of developed bioinformatics software, especially porting efforts, can help to address issues with system and platform variations. (Steinberg, 2003) asked bioinformatics engineers to create an online community in which they could share not only data but also developed software and stressed the value of reusing software by encouraging bioinformatics software be made open-source. Following Steinberg’s research, great standardization efforts have been developed and deployed. Standardized API documentation using OpenAPI (Mulligan, 2008) and BrAPI (Selby et al., 2019), ontologies for annotating bio tools (*e.g*. EDAM (Ison et al., 2013)), workflow systems including a great number in life sciences ranging from analytic platforms like KNIME (Fillbrunn et al., 2017) to Jupyter Notebooks (Kluyver et al., 2016), command-line workflow tools like Nextflow (Di Tommaso et al., 2017), and dependency systems like SnakeMake (Köster & Rahmann, 2012) have all aided the bioinformatics community. Additionally, containers such as Bioboxes (Belmann et al., 2015) and BioContainers (da Veiga Leprevost et al., 2017), packagers such as Bioconda (Grüning et al., 2018), and distributions (https://wiki.debian.org/DebianScience/Biology) have proven very useful. The Common Workflow Language (http://www.commonwl.org) is an effort primarily driven by the life science community. All of these efforts have helped to tackle the challenge of integration for scientific bioinformatics software.

With integration and reproducibility of bioinformatics software being so closely tied when performing data-driven research, embracing open approaches to managing software development have proven highly beneficial. It enables bioinformatics engineers to build on the work of other works while also allowing others to shape their efforts and help solve their collective problems. This leads to the establishment the software with greater longevity, more effective problem solving, and longer sustainability (Ivie & Thain, 2018).

## Conclusion

This study explores several areas of software development that pose both challenges and possibilities for the development of scientific bioinformatics software, which include requirements gathering, documentation, testing, and integration. When it comes to bioinformatics engineers mastering such skills, many cultural factors must be considered, including funding patterns that reward new development and preclude reuse as well as lack of sustainability of third-party codes and reluctant support for their adoption. Bioinformatics engineers are often isolated or alone in research groups. They may not be afforded the time or capacity to incorporate outside software nor prepare their own software for broader usage, possibly as a result of project or PI requirements. To really understand the relationship between bioinformatics and software engineering requires understanding the drivers.

Bioinformatics is still in its infancy compared with the majority of other scientific disciplines. The combination of mathematics, statistics, and computation in a cross-disciplinary fashion in conjunction with numerous fields is a driving factor in adopting bioinformatics practices. The objective is to merge extensive knowledge of both computation and biology into one brain in order to appropriately address the complexities of life science datasets and problems with effective software solutions. This can prove costly and unrealistic in many scenarios. Software engineering practices for producing valid, maintainable, and sustainable solutions, while abstract in their nature, can prove challenging to apply directly to bioinformatics research due to factors inherent within the life sciences and bioinformatics themselves. This basically necessitates that bioinformatics engineers incorporate and adapt software engineering skills and principles into their skillsets rather than attempting to collaborate with software engineers lacking expertise in bioinformatics. However, when aspects of project management of the development of bioinformatics software are considered, it is possible for bioinformatics engineers to collaborate openly with one another and professional software engineers to facilitate and enhance their efforts. These efforts may be the key to producing higher-quality scientific bioinformatics software for the future through the structured development of more robust software and the reduced recreation of the wheel. Bioinformatics engineers can reap the benefits of applying software engineering principles in terms of the efficiency and effectiveness of their works by keeping their collective software development efforts DRY (don’t repeat yourself).

## Supplemental Information

10.7717/peerj-cs.839/supp-1 Supplemental Information 1 PRISMA Flowchart.

## Additional Information and Declarations

### Data Availability

The following information was supplied regarding data availability: This is a literature review.
