# Supplementary figures and images for: Improving bioinformatics software quality through incorporation of software engineering practices
Source: PeerJ Comput Sci. 2022 Jan 5;8:e839. doi: 10.7717/peerj-cs.839 (PMC8771759; doi:10.7717/peerj-cs.839)

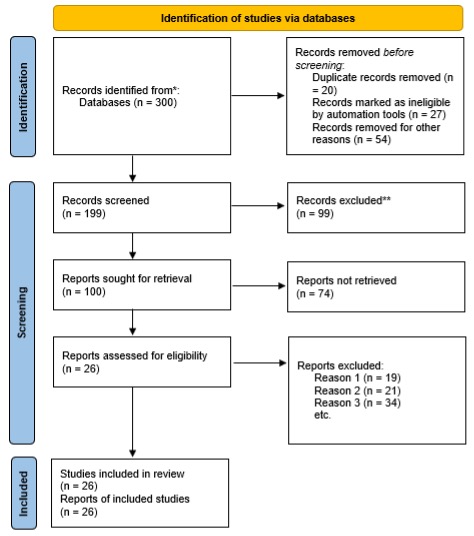

Supplement: Supplemental Information 1 [file peerj-cs-08-839-s001.jpg]
